# The prevalence of thyroid dysfunction and autoimmune thyroid disease in patients with rheumatoid arthritis

**DOI:** 10.1186/s41927-022-00293-9

**Published:** 2022-10-24

**Authors:** Mahsa Bagherzadeh-Fard, Mohammad Amin Yazdanifar, Mohammad Aghaali, Maryam Masoumi

**Affiliations:** Qom University of Medical Sciences, Qom, Iran

**Keywords:** Rheumatoid arthritis, RA, Thyroid dysfunction, Anti-thyroid autoantibody, Anti-TPO, Autoimmune thyroid disease, AITD, Thyroid hormone abnormality

## Abstract

**Background:**

Rheumatoid arthritis (RA) is one of the most common chronic non-organ-specific autoimmune diseases; meanwhile, autoimmune thyroid disease (AITD) is the most common organ-specific autoimmune disease that can lead to hypo or hyperthyroidism. Although the etiology of both diseases is complex with a combination of genetic and environmental factors, there are overlaps in genes contributing to the pathogenesis of both diseases. Numerous studies found a correlation between thyroid abnormality and RA in different populations, yet some didn’t. This study is aimed to evaluate the prevalence of thyroid dysfunction, AITD, and anti-thyroid peroxidase (anti-TPO) positively in Iranian patients with RA.

**Methods:**

A total of 250 RA patients and 248 patients with non-inflammatory rheumatologic disease were included in this case–control study. All participants underwent complete clinical and laboratory assessments. Participants were also assessed for thyroid function testing, including anti-TPO antibodies.

**Results:**

Thyroid dysfunction was twice as common in RA patients as in controls (OR = 2.16; P-value > 0.001). Overt hypothyroidism was the most common thyroid dysfunction among RA patients (58 out of 84). Anti-TPO positivity was also significantly more common in RA compared with controls (OR = 2.65; P-value > 0.001). The proportion of controls and RA patients with AITD were 9 and 21.5%, respectively. AITD was found 2.8 times more common in RA group than controls (OR = 2.77; P-value > 0.001).

**Conclusion:**

It was demonstrated that RA was an independent factor associated with thyroid dysfunction and AITD.

**Supplementary Information:**

The online version contains supplementary material available at 10.1186/s41927-022-00293-9.

## Background

Rheumatoid arthritis (RA) is a chronic autoimmune systemic inflammatory disease characterized by symmetrical involvement of the peripheral small joints. The overall prevalence of RA is 0.5–1% of general population and RA is twice or threefold more common among females compared to males. RA is a multifactorial disease which both genetic and environmental factors play an important role in disease’s pathogenesis [1]. RA disease can also interfere with different aspects of lifestyle and physical activity leading to increase disability [2, 3]. Although RA mainly damages joints, it is a systemic disease, and almost 40.6% of the patients have extra-articular manifestations [4]. Extra-articular involvement of RA consists of cutaneous, cardiovascular, pulmonary, gastrointestinal, ocular, renal, and neurologic presentations [5].

Thyroid dysfunction is mainly divided into hypothyroidism (insufficiency of thyroid hormones) and hyperthyroidism (excess of thyroid hormones). Each of these can be further subdivided as overt and subclinical thyroid dysfunction [6]. Autoimmune thyroid disease (AITD) is the most prevalent organ-specific autoimmune disorder with a frequency of 5% within the public in general [7]. AITD is closely linked to, rheumatologic disease, including Sjögren syndrome, Systemic Lupus Erythematosus, and Systemic sclerosis. RA and AITD connection were not fully investigated [8, 9].

Several studies determined various ranges of frequency of thyroid dysfunction, AITD, and anti-thyroid autoantibody like anti-thyroid peroxidase (anti-TPO) in RA patients in different places [10]. Although the mechanism of RA and AITD association is not fully understood yet, several studies showed autoimmunity as a vital factor in the pathogenesis of both diseases [7]. Some genes were also discovered to be key factors in both diseases’ development such as STAT4, HLA-DRB1, and vitamin D receptor [11]. Considering the similar pathogenesis in both RA and thyroid abnormality which can coexist in some patients, and insufficient research on this matter, especially not accounting for racial diversity as a potential risk factor, this study aimed to determine the association between RA and thyroid dysfunction, AITD, as well as anti-TPO positivity in Iranian population.

## Methods

### Study design

This observational, case–control study was conducted at Shahid Beheshti tertiary care center, Qom, Iran, from April to October 2021. The protocol was approved by the ethics committee of Qom University of Medical Sciences and informed consent, according to the Declaration of Helsinki, was taken from all participants before enrollment.

### Participants

#### Cases

Two hundred fifty RA patients who presented to the outpatient clinic of our center were consecutively included. All patients were aged between 18 and 85 and met the American College of Rheumatology and the European League against Rheumatism (ACR/EULAR) 2010 criteria for rheumatoid arthritis [12]. Patients with chronic liver or renal disease, diabetes mellitus, concomitant infection, malignancy, any collagen vascular disease other than RA, pregnant females or patients consuming medication that can lead to thyroid dysfunction (e.g., interferon-α, dopamine agonists, amiodarone, anticonvulsant drugs, somatostatin analogs, and lithium) were excluded.

#### Controls

Two hundred forty-eight individuals were consecutively selected from patients with non-inflammatory rheumatologic disease including osteoarthritis, neck or back pain, chondromalacia, spinal canal stenosis, periarthritis (e.g., Tennis elbow, De Quervain`s tenosynovitis and, bursitis). None of them meet the exclusion criteria mentioned above. Our control group had almost the same demographic features as our patient group.

### Clinical assessments

The data was collected by reviewing medical records of patients, interviews, and physical examination. Participants’ demographic features were also taken into consideration. They were also evaluated for prior history of thyroid hormone disturbance or thyroid drug consumption to assess previous thyroid dysfunction. visual analog scale (VAS) and DAS-28-ESR were also assessed [13]. Laboratory investigations, including quantitative C-reactive protein (CRP) titer based on Biotec method, and erythrocyte sedimentation rate (ESR) estimated by using the Westergren method was tested in all participants. Anti-cyclic-citrullinated peptide antibodies (anti-CCP) and anti-mutated-citrullinated vimentin autoantibodies (anti-MCV) using an enzyme-linked immunosorbent assay (ELISA) method, and rheumatoid factor (RF) only were also checked for RA patients. CRP, ESR, Anti-CCP and anti-MCV were positive for level of more than 6 mg/l, 20 mm/h, 5 U/ml, and 20 U/ml, respectively. Thyroid function tests were performed using chemiluminescent immunoassay (CLIA) method with a normal range of 0.3–3.6 mIU/L for thyroid stimulating hormone (TSH), 0.7–1.8 ng/dl for free thyroxin (FT4), 2.57–4.43 pg/mL for free triiodothyronine (FT3). Thyroid dysfunction was diagnosed based on evaluation of thyroid hormones [6]:

- Overt hypothyroidism: TSH > 3.6 and FT4 < 0.7
- Subclinical hypothyroidism: TSH > 3.6 with normal range of FT4
- Overt hyperthyroidism: TSH < 0.3 and FT4 > 1.8 or FT3 > 4.43
- Subclinical hyperthyroidism: TSH < 0.3 with normal range of FT4 and FT3
- Euthyroid: normal range of TSH and FT4

Anti-TPO antibody was also determined using radioimmunoassay with an optimum cut-off level of 50 IU/mL. AITD was defined by positive anti-TPO and the presence or history of thyroid dysfunction [14].

### Statistical analyses

Analysis of data was carried out by IBM SPSS Statistics version 26. Before any analysis was done, normality was checked using the Kolmogorov–Smirnov test, Q–Q, and P-P plots in continuous variables. Quantitative data with normal distributions including age, TSH and FT3 were described as mean ± standard deviation (SD) and all other data with non-normal distributions were reported as median (25–75th percentile). The difference of variables between cases and controls was assessed by using the Mann–Whitney U test for non-normally distributed data or the Student’s T-test for normally distributed data. Qualitative variables like laboratory test positivity and the proportion of thyroid abnormality were described in number and percentage. The comparisons of categorical variables between cases and controls were also determined using the chi-square test and odds ratio. When the chi-square test was inappropriate, the Fisher exact test was applied. Multivariant logistic regression was used to determine independent factors associated with thyroid dysfunction and AITD. The odds ratio (OR), and 95% confidence interval (CI) were presented as well. In all tests, P-value < 0.05 was considered statistically significant and it was non-significant if the P-value was > 0.05.

## Results

This study included 250 patients with RA disease as cases and 248 patients with a non-inflammatory rheumatological disease as controls. Fifty-three (21.4%) individuals of controls suffer from back or/and neck pain, 111 (44.8%) from osteoarthritis, 52 (21.0%) from periarthritis, 28 (11.3%) from chondromalacia, and 3 (1.2%) from spinal canal stenosis.

The results of clinical characteristics of cases and controls were shown and compared in Table 1. The number of participants with ESR and CRP positive were 25 (10.2%) and 10 (4.1%) in controls as well as 111 (45.5%) and 103 (42.6%) in cases. Anti-CCP and anti-MCV were also positive in 142 (56.8%) and 133 (53.4%) of cases. It was demonstrated that RA patients have a significantly higher level of ESR, CRP, TSH, FT3, FT4 and, anti-TPO compared with the control group.

**Table 1:** Comparisons between cases and controls in terms of Clinical characteristics using Student’s T-test, Mann Whitney, or odds ratio

Clinical characteristics	Controls (n = 248) Median (25th–75th percentile), mean ± SD or n (%)	Cases (n = 250) Median (25th–75th percentile), mean ± SD or n (%)	z/t/OR	P-value
Age (year)	51.0 ± 12.6	52.6 ± 12.7	− 1.31	0.190
Females	223 (89.9%)	211 (84.4%)	1.65	0.081
Duration of RA disease (year)		5.0 (3.0–10.0)		
Tender joint count		4.0 (0.0–13.0)		
Swollen joint count		3.0 (0.0–11.0)		
VAS		70.0 (0.0–100.0)		
DAS-28-ESR		4.9 (1.9–6.6)		
*Laboratory finding*				
ESR (mm/h)	10.0 (6.0–14.2)	18.5 (10.0–41.0)	− 8.9	< 0.001
CRP (mg/l)	3.0 (2.5–4.0)	5.0 (2.0–18.0)	− 6.6	< 0.001
TSH (mIU/L)	2.3 ± 2.4	2.9 ± 2.9	− 2.3	0.024
FT3 (pg/mL)	3.1 ± 1.3	3.9 ± 1.6	− 5.9	< 0.001
FT4 (ng/dl)	1.0 (0.9–1.3)	1.2 (1.0–1.6)	− 6.0	< 0.001
Anti-TPO (IU/ml)	8.9 (5.0–25.0)	17.3 (6.4–157.0)	− 4.6	< 0.001
Anti-CCP (U/ml)		15.9 (2.2–185.5)		
Anti-MCV (U/ml)		28.0 (10.0–311.5)		
RF positive		112 (45.2%)		
*RA Medications*				
Prednisolone		228 (91.2%)		
Methotrexate		222 (88.8%)		
Hydroxychloroquine		151 (60.4%)		
Sulfasalazine		62 (24.8%)		
Leflunomide		61 (24.4%)		
Etanercept		7 (2.8%)		
Adalimumab		4 (1.6%)		

*RA* rheumatoid arthritis, *VAS* Visual Analogue Scale, *DAS-28-ESR* disease activity score 28 for RA with ESR, *ESR* erythrocyte sedimentation rate, *CRP* C-reactive protein, *TSH* thyroid stimulating hormone, *FT4* free thyroxin, *FT3* free triiodothyronine, *anti-TPO* anti-thyroid peroxidase, *anti-CCP* anti-cyclic-citrullinated peptide antibodies, *anti-MCV* anti-mutated-citrullinated vimentin autoantibodies, *RF* rheumatoid factor. Using Student’s T-test for TSH and FT3 and Mann–Whitney for others. P-value < 0.05 was considered significant

The number of controls and cases with thyroid dysfunction were 47 (19.2%) and 84 (33.9%), respectively, in which 28 (11.3%) controls and 60 (24.2%) cases have prior history of thyroid dysfunction. A significant association was found between RA disease and thyroid dysfunction which thyroid dysfunction was twice as common in RA patients as in controls (OR = 2.16, 95% CI: 1.43–3.26; P-value > 0.001). As presented in Table 2, the most common thyroid dysfunction was overt hypothyroidism. Only overt hypothyroidism was associated with RA disease and there was no significant association between other forms of thyroid dysfunction and RA.

**Table 2:** Frequency of thyroid dysfunctions among cases and controls

Thyroid Dysfunctions	Controls (n = 245)* [n (%)]	Cases (n = 248)* [n (%)]	Unadjusted OR (95% CI)	P-value
Euthyroid	198 (80.82%)	164 (66.13%)	0.46 (0.30–0.70)	> 0.001
Overt hypothyroidism	29 (11.83%)	58 (23.39%)	2.27 (1.39–3.69)	0.001
Subclinical hypothyroidism	16 (6.53%)	22 (8.87%)	1.39 (0.71–2.72)	0.399
Overt hyperthyroidism	1 (0.41%)	4 (1.61%)	4.00 (0.44–36.04)	0.372
Subclinical hyperthyroidism	1 (0.41%)	0 (0%)	0.99 (0.98–1.00)	0.497

CI: confidence interval, OR: odds ratio. P-value < 0.05 was considered significant

*Three were three and two patients with missing data in cases and controls, respectably

**Fig. 1:**
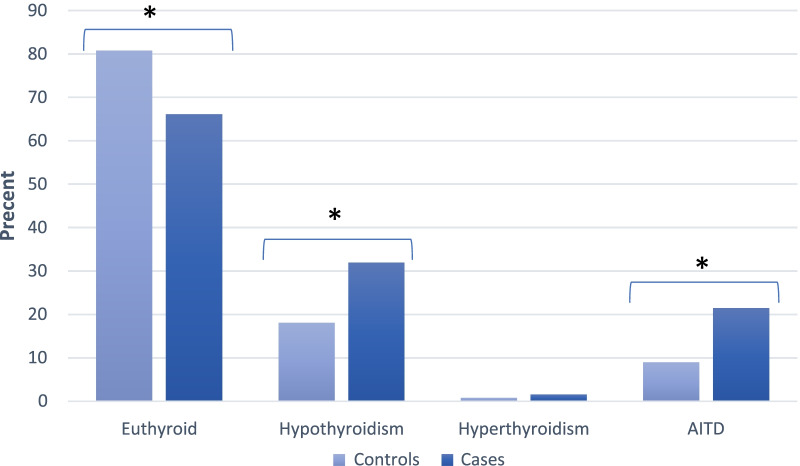
Thyroid dysfunction and Autoimmune thyroid disease (AITD) disparity among cases and controls. * The difference was statistically significant with the P-value > 0.001

**Fig. 2:**
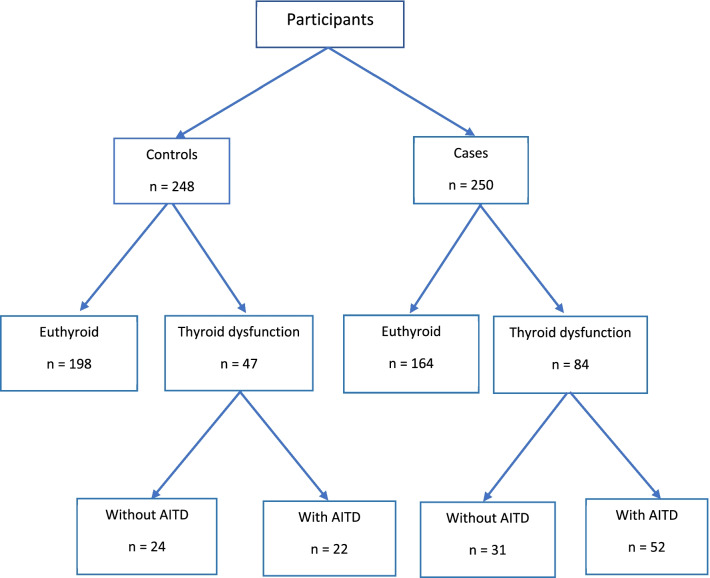
Presence or absence of autoimmune thyroid disease (AITD) in patients with thyroid dysfunction

Anti-TPO was positive in 37 (15.0%) controls, and 77 (32.0%) cases and it was significantly more common in RA patients (OR = 2.65, 95% CI: 1.70–4.13; P-value > 0.001). The frequency of AITD was 22 (9.0%) and 52 (21.5%) in controls and cases, respectively, therefore it was found AITD was 2.77 times more common in RA patients than that in controls (OR = 2.77, 95% CI: 1.62–4.73; P-value > 0.001). The proportion of overt/subclinical hypothyroidism and hyperthyroidism, as well as AITD among controls and cases, is shown in Fig. 1. The presence of AITD in thyroid dysfunction for both groups of controls and RA patients was demonstrated in Fig. 2.

As it is shown in Table 3, RA is an independent factor associated with both thyroid dysfunctions and AITD; however, gender and age are not significantly associated with each disease.

**Table 3:** Binary logistic regression analysis evaluating independent predictors associated with thyroid dysfunction and AITD

Variables	Thyroid dysfunction		AITD	
	Adjusted OR (95% CI)	P	Adjusted OR (95% CI)	P
Gender (female sex)	0.84 (0.46–1.52)	0.561	0.71 (0.35–145)	0.354
Age (year)	1.00 (0.98–1.02)	0.607	1.01 (0.99–1.03)	0.310
RA	2.11 (1.39–3.20)	> 0.001	2.79 (1.63–4.77)	> 0.001

*RA* rheumatoid arthritis, *OR* odds ratio, *CI* confidence interval. P-value < 0.05 was considered significant

## Discussion

The relationship between RA and thyroid dysfunction has been taken into account since the 1960s when an association between RA and Hashimoto’s thyroiditis based on the common underlying role of autoimmunity was found [15, 16]. The prevalence of thyroid dysfunction with or without AITD is estimated to be between 6 and 33.8% in RA patients [17]. However, it is worthy of mention that thyroid dysfunction, AITD, and anti-TPO positivity prevalence varies among RA patients based on geographical locations as instance prevalence of AITD among RA patients can range from 0.5% in Morocco [18] to 27% in Slovakia [19]; and anti-TPO differ from 5% [20] in Egypt to 37% in Italy [21].

Since in early research the control group was absent, some recent studies have investigated the association between thyroid hormone dysfunction and AITD in RA patients [22]. Although Sara McCoy et al. found no difference in prevalence or development of hypothyroidism in RA patients, Prakash Joshi et al., in a survey enrolling 52 RA patients, estimated hypothyroidism was 3.5 times more prevalent compared with the general population [23, 24]. Since a firm consensus is not reached on this matter and the association also was not well studied well in the Iranian population, this case–control study enrolling 250 RA and 248 non-inflammatory rheumatological patients have been designed to investigate this issue. This study demonstrated a significant association between thyroid dysfunction and RA which was twice as common in RA patients as in controls. The prevalence of patients with thyroid dysfunction were 19.2% in controls and 33.9% in cases which overt hypothyroidism was the most prevalent form including 11.8% and 23.4% of thyroid dysfunction. Like most of the previous research, we found significant association with hypothyroidism but not for hyperthyroidism. Nevertheless, Qian Li et al. found both hypo and hyperthyroidism were significantly prevalent in RA patients than controls among the Chinese population [25], and this discrepancy may be due to low prevalence of hyperthyroidism in our participants. It should also be mentioned that several studies only consider overt hypothyroidism and did not include the subclinical stage in their evaluation, yet some studies included both stages. In most of these studies, overt hypothyroidism was the most common thyroid dysfunction [11, 25–27]; however, several of them showed subclinical hypothyroidism as the most common one [17] while others found no significant difference between subgroups [28]. We concluded in this study that RA only associated with overt hypothyroidism, 2.3 times more common among RA patients than controls, and no significant association between RA and subclinical form was observed.

Moreover, recent studies have paid more attention to the prevalence of anti-thyroid antibodies positivity and AITD in RA patients. Xi-Feng Pan et al., in a meta-analysis, estimated that the presence of anti-TPO is 2.3 times more common in RA patients compared with healthy individuals. Although anti-thyroid autoantibodies were positively associated with RA disease in Asian and African populations, no significant association was detected in most of the studies conducted in America and Europe. The reason for this contrast might lie in geographical genetic, and environmental differences or preliminary studies to find its real association [29]. In this study, we also estimated that the prevalence of anti-TPO positivity was 15.0% of controls and 32.0% of cases and it was 2.65 times greater in RA patients compared with controls.

The association between AITD, an organ-specific autoimmune disease, and systematic autoimmune diseases like systemic lupus erythematous, primary Sjögren’s syndrome, and especially RA was shown in several studies which can be partly explained by the similar underlying autoimmune pathogenesis and possible genetic susceptibility [22]. Despite the finding that AITD was more common among RA patients compared to healthy individuals, some studies found this difference to be not statistically significant [17]. Whereas in our analysis, AITD was 2.5 times more prevalent in RA patients compared with controls which was statistically significant (19.7 vs. 8.6%).

Although seronegative thyroiditis, which is characterized by thyroid dysfunction without positive tests for thyroid autoantibodies and a hypoechoic pattern of the thyroid parenchyma at ultrasound, was not included in this study, it does not seem to have a significant impact on our analysis because of low prevalence of this form of thyroiditis (~ 5%) [30]. Nonetheless, this research is subject to several limitations such as lack of measuring other kinds of anti-thyroid antibodies and detailed factors affecting the prevalence of thyroid abnormality in RA patients, for instance body mass index, smoking (although it is notable that most of the enrolled patients were middle-aged women and smoking is scarce in this demographic of Iranian population), lifestyle, genetic background, and so on; hence, it leaves room for further studies.

In conclusion, this study demonstrated that RA disease was an independent covariant for thyroid dysfunction (especially for overt hypothyroidism), presence of anti-TPO positivity, and AITD among the Iranian population like several other populations.

## Supplementary Information

**Additional file 1.** It consist of all data generated during this study, which includes age, gender, duration of RA, history of thyroid disease and its subtypes, tender and swollen joint count, VAS, History of medication used for RA treatment, lab studies such as TSH, FT3, FT4, anti-CCP, anti-MCV, anti-TPO, RF, ESR, CRP and DAS-28.

## Data Availability

All data generated or analyzed during this study are included in this published article and its additional file (Additional file 1).

## Abbreviations

RA: Rheumatoid arthritis
AITD: Autoimmune thyroid disease
anti-TPO: Anti-thyroid peroxidase
ACR/EULAR: American College of Rheumatology and the European League against Rheumatism
CRP: C-reactive protein
ESR: Erythrocyte sedimentation rate
anti-CCP: Anti-cyclic-citrullinated peptide antibodies
anti-MCV: Anti-mutated-citrullinated vimentin autoantibodies
ELISA: Enzyme-linked immunosorbent assay
RF: Rheumatoid factor
CLIA: Chemiluminescent immunoassay
TSH: Thyroid stimulating hormone
FT4: Free thyroxin
FT3: Free triiodothyronine
SD: Standard deviation
OR: Odds ratio
CI: Confidence interval

## References

[1] Smolen JS (2018). Rheumatoid arthritis. Nat Rev Dis Primers.

[2] Tabaraii R (2021). Association of lifestyle and disease characteristics with self-rated wellness/health score in patients with rheumatoid arthritis. BMC Rheumatol.

[3] Bala SV (2021). Reported disability in relation to observed activity limitation, grip strength and physical function in women and men with rheumatoid arthritis. BMC Rheumatol.

[4] Turesson C, O’Fallon WM, Crowson CS, Gabriel SE, Matteson EL (2003). Extraarticular disease manifestations in rheumatoid arthritis: incidence trends and risk factors over 46 years. Ann Rheum Dis.

[5] Young A, Koduri G (2007). Extra-articular manifestations and complications of rheumatoid arthritis. Best Pract Res Clin Rheumatol.

[6] Criteria for thyroid abnormalities according to the Dutch national healthcare consensus committee. http://www.dk.cvz.nl

[7] Lazúrová I (2014). Autoimmune thyroid disease and rheumatoid arthritis: relationship and the role of genetics. Immunol Res.

[8] Bourji K (2015). Rheumatic and autoimmune thyroid disorders: a causal or casual relationship?. Autoimmun Rev.

[9] Lazúrová I, Benhatchi K (2012). Autoimmune thyroid diseases and nonorgan-specific autoimmunity. Pol Arch Med Wewn.

[10] Mahagna H (2018). Rheumatoid arthritis and thyroid dysfunction: a cross-sectional study and a review of the literature. Best Pract Res Clin Rheumatol.

[11] Elattar EA, Younes TB, Mobasher SA (2014). Hypothyroidism in patients with rheumatoid arthritis and its relation to disease activity. Egypt Rheumatol Rehabil.

[12] Aletaha D, Neogi T, Silman A, Funovits J, Felson D, Bingham C (2010). 2010 rheumatoid arthritis classification criteria: an american college of rheumatology/european league against rheumatism collaborative initiative. Ann Rheum Dis.

[13] Prevoo ML, Van'T Hof M, Kuper HH, Van Leeuwen MA, Van De Putte LB, Van Riel PL (1995). Modified disease activity scores that include twenty-eight-joint counts development and validation in a prospective longitudinal study of patients with rheumatoid arthritis. Arthr Rheum Off J Am Coll Rheumatol.

[14] Cárdenas Roldán J (2012). Autoimmune thyroid disease in rheumatoid arthritis: a global perspective. Arthritis.

[15] Hijmans W (1961). Serological overlap between lupus erythematosus, rheumatoid arthritis, and thyroid auto-immune disease. Bri Med J.

[16] Becker KL, Ferguson RH, McConahey WM (1963). The connective-tissue diseases and symptoms associated with Hashimoto's thyroiditis. New Engl J Med.

[17] Przygodzka M, Filipowicz-Sosnowska A (2009). Prevalence of thyroid diseases and antithyroid antibodies in women with rheumatoid arthritis. Pol Arch Med Wewn.

[18] Benamour S (1992). Rheumatoid arthritis in Morocco. Apropos of 404 observations. Revue du Rhumatisme et des Maladies Osteo-Articulaires.

[19] Lazúrová I (2009). Autoimmune thyroid disease and autoimmune rheumatic disorders. Ann N Y Acad Sci.

[20] El-Sherif WT (2004). Thyroid disorders and autoantibodies in systemic lupus erythematosus and rheumatoid arthritis patients. Egypt J Immunol.

[21] Atzeni F (2008). Anti-thyroid antibodies and thyroid dysfunction in rheumatoid arthritis: prevalence and clinical value. Autoimmunity.

[22] Conigliaro P (2020). Autoimmune thyroid disorders and rheumatoid arthritis: a bidirectional interplay. Autoimmun Rev.

[23] McCOY SS (2012). Hypothyroidism as a risk factor for development of cardiovascular disease in patients with rheumatoid arthritis. J Rheumatol.

[24] Joshi P (2017). Prevalence of hypothyroidism in rheumatoid arthritis and its correlation with disease activity. Trop Doct.

[25] Li Q (2018). Increased risk of thyroid dysfunction among patients with rheumatoid arthritis. Front Endocrinol (Lausanne).

[26] Emamifar A, Hangaard J, Jensen Hansen IM (2017). Thyroid disorders in patients with newly diagnosed rheumatoid arthritis is associated with poor initial treatment response evaluated by disease activity score in 28 joints-C-reactive protein (DAS28-CRP): an observational cohort study. Med (Baltimore).

[27] Saqre IM (2019). Autoimmune thyroid disease in Egyptian patients with rheumatoid arthritis. Egypt Rheumatol.

[28] Chen YL (2018). Joint damage is amplified in rheumatoid arthritis patients with positive thyroid autoantibodies. PeerJ.

[29] Pan XF, Gu JQ, Shan ZY (2015). Increased risk of thyroid autoimmunity in rheumatoid arthritis: a systematic review and meta-analysis. Endocrine.

[30] Takamatsu J, Yoshida S, Yokozawa T, Hirai K, Kuma K, Ohsawa N, Hosoya T (1998). Correlation of antithyroglobulin and antithyroid-peroxidase antibody profiles with clinical and ultrasound characteristics of chronic thyroiditis. Thyroid.

